# Research on a New Localized Induction Heating Process for Hot Stamping Steel Blanks

**DOI:** 10.3390/ma12071024

**Published:** 2019-03-28

**Authors:** Li Bao, Jingqi Chen, Qi Li, Yu Gu, Jian Wu, Weijie Liu

**Affiliations:** 1 School of Material Science and Engineering, Northeastern University, Shenyang 110006, Liaoning, China; lwjxs2012@163.com (L.B.); chenjq@163.com (J.C.); liqi.hnxc@gmail.com (Q.L.); cicielf1212@hotmail.com (Y.G.); wujianneu@163.com (J.W.); 2 School of Mechanical and Electrical Engineering, Qiqihar University, Qiqihar 161006, Heilongjiang, China

**Keywords:** localized inductive heating, hot stamping steel blanks, tailored properties, magnetizer

## Abstract

Localized induction heating with one magnetizer was experimentally analyzed in order to investigate the altering effect of the magnetizer on the magnetic field. A 22MnB5 blank for tailored property was locally heated to produce the parts of a car body in white, such as the B-pillars. A lower-temperature region with a temperature in the two-phase zone and a full-austenitic high-temperature region were formed on the steel blank after 30 s. After water-quenching, the mixture microstructure (F + M) and 100% fine-grained lath martensite were obtained from the lower- and high-temperature regions, respectively. Moreover, the ultimate tensile stress (UTS) of the parts from the lower- and high-temperature regions was 977 and 1698 MPa, respectively, whereas the total elongations were 17.5% and 14.5%, respectively. Compared with the parts obtained by conventional furnace heating–water quenching (UTS: 1554 MPa, total elongation: 12%), the as-quenched phase developed a tensile strength over 100 MPa greater and a higher ductility. Thus, the new heating process can be a good foundation in subsequent experiments to arbitrarily tailor the designable low-strength zone with a higher ductility by using magnetizers.

## 1. Introduction

One of the significant technical achievements during the last few decades in the automobile industry was the use of lightweight design to reduce the fuel consumption and greenhouse gas emission of vehicles. When the weight of a vehicle is reduced by 10%, its fuel consumption and emission will decline by 8–10% and 4–6%, respectively [1]. Although low-density materials, such as aluminum, magnesium, and/or carbon fiber-reinforced polymers, can be used to make lightweight automobile parts, their costs are considerably higher than steels [2]. As a result, steels, especially the advanced high strength steel blanks (AHSS), remain the most important materials for stamping automobile parts. Hot stamping, which transforms the microstructure of steel blanks into the stronger martensite phase while being formed, has become a famous industrial process for manufacturing lightweight parts in recent years. The applications of the above lightweight parts are important technical advances in the automobile industry, but the general invariant mechanical properties limit the maximization of the overall performances, which is a limitation that should be overcome. Obviously, it is a tailorable rather than a constant distribution of mechanical properties that facilitates the optimization of the overall performance of a stamped part, including its weight, efficiency, passenger safety, and cost [3]. For example, a B-pillar with higher strength in the upper region than in the bottom region may better protect passengers and maximize energy absorption during a vehicle crash. Such blanks with tailored mechanical properties are usually fabricated using laser welding that joins two or more tailored metal blanks, which all have different strength, thickness, or material. For example, thickness-difference blanks with tailored properties can also be produced by special rolling mails with sectional roll gaps, each of which has a different gauge [4]. Furthermore, equal-thickness and homogenous steel blanks with tailored properties can also be obtained if their microstructure can be changed locally during hot stamping. 

In detail, three strategies including specific austenitization, specific cooling, and specific annealing will be introduced [5]. An absorption component which was brought into contact with the blank to suppress its austenitization was proposed [6], and then the ductile zones were formed. Two methods including spray cools and masked austenitization to generate the parts with tailored properties were presented [3]. The heated and cooled tool by varying cooling rates to produce the parts with tailored properties was designed [7]. As for specific annealing, localized tempering of the transformed martensite phase to generate ductile properties was put forward [8]. Although all the proposed techniques are capable of creating a tailored property effect by forming a mixed microstructure of martensite (hard phase) and ferrite or tempered martensite (soft phase), they are either too complicated or too time consuming to be operated and too expensive, so none of them have been industrially applied. In addition, all known parts with tailored property-related technologies, either applied in industries or under development in the laboratory, lack flexibility in designing and tailoring the property distributions on blanks, which may weaken the effort of overall performance optimization for the lightweight parts.

To find a practical process to produce hot stamped parts with finely designable tailored properties, our solution is based on induction heating, which is not a new technology and has been widely used in parts such as crankshafts, sprockets, steel tubes, and gears. However, it is currently suitable for the overall heating of small parts and the surface heating of large parts. There are many theoretical, experimental, and simulation studies on induction heating. As for the blanks for hot stamping, induction heating was first applied on 22MnB5 by a combination of a longitudinal and a transverse magnetic field and then quenched to generate the uniform martensite microstructure [9]. As an important part, the magnetizer played an important role in the induction heating. For instance, a working coil with magnetic flux concentrators to enhance the uniform heating effect on the barrel was applied [10]. Moreover, the magnetizer was used to shield the magnetic flux line to develop the uniform effect on the nonplanar mold surface [11]. With the same purpose, the effect of different magnetizer dimensions on the homogeneous heating of workpieces was investigated [12]. These induction heating methods developed the temperature distribution of the heated workpiece more uniformly with or without the magnetizer, however, they have not been used for a stamped part with tailored properties.

In this article, a new localized induction heating method was proposed. Specifically, the uniform alternating electromagnetic field generated by a flattened solenoid induction coil can be locally altered by a magnetizer piece, so that the eddy current strength in the steel blank placed in the coil can be locally weakened, leading to the formation of ferrite ponds surrounded by austenite phases. Thus, after stamping by a water-cooled mold, the austenite in the blank transformed into hard martensite and the ferrite remained. Finally, a stamped part with tailored properties was produced.

## 2. Experimental Method

### 2.1. Material 

The 22MnB5 blanks with a nominal thickness of 3.5 mm (Baogang Group) were used here. The chemical composition of the material is shown in Table 1. The steel has a Curie temperature of 760 °C (T_C_), below which it is ferromagnetic and above which the loss of ferromagnetic properties will be revealed. Moreover, the basic information of the steel blanks, the inductor, and the magnetizer used in localized induction heating experiments is shown in Table 2.

### 2.2. Localized Induction Heating Method

An induction coil of a high-frequency inductor was composed of six parallel copper tubes with a spacing of 10 mm to form a flat solenoid. The steel blanks were placed in an internal rectangular space, which was formed by the upper and lower copper tubes and was about 130 mm in length, 200 mm in width, and 15 mm in height. Two kinds of induction heating experiments were carried out through the inductor. The first one was uniform induction heating, and the second experiment was localized induction heating, which was based on the first one. In detail, the dimensions are shown in Table 2. Because the dimension and shape of the magnetizer may have different effects on the temperature field of the steel blank, they will be investigated in a following study. Therefore, this paper only studies the localized heating with a certain size of magnetizer piece as an example.

**Figure 1:**
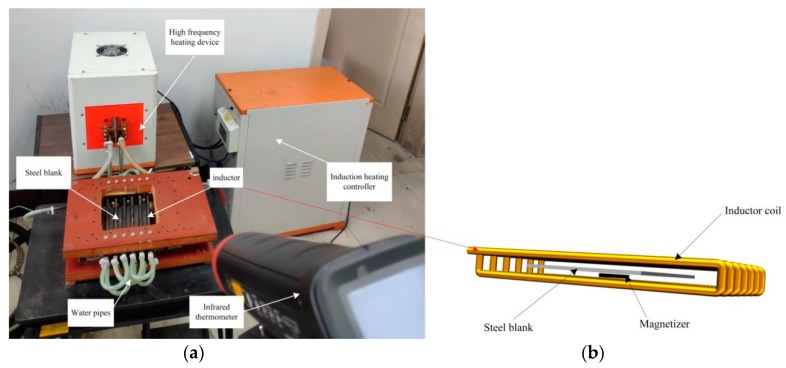
Localized induction heating system. (**a**) Experimental setup; and (**b**) schematic diagram of localized induction heating model.

This solenoid induction coil can produce a uniform and stable alternating electromagnetic field, which can inductively heat up the entire body of a steel blank to a uniform temperature. It was proved that its entire body was heated up quickly and evenly from room temperature to 915–925 °C in 30 s. When a magnetizer piece was placed between the lower layer coils and the center of the steel blank, the inductive heating device became ready for localized heating. The physical model of localized induction heating consisted of a solenoid coil (yellow), a magnetizer (black), and a steel blank (gray, Figure 1b). After 30 s of localized induction heating, the steel blank was quickly transferred for water quenching. Since the quenching time should last at least 60 s [13] and in order to ensure complete quenching, we set the quenching time at 120 s.

The induction heating system, which consisted of an inductive coil, a steel blank, a magnetizer, an induction heating device, a controller, and a temperature measuring device, is also depicted in Figure 1. Since the temperature of the workpiece under the induction heating is mostly measured by the infrared measurement method, it can be noticed that a square-shaped window was made in the center of the top insulation plate where the upper coils were mounted, through which the heating up history of the blank could be recorded using an infrared thermometer (ε = 0.7, precision: ±2 °C, 3i Plus, Raynger, Santa Cruz, CA, USA). Meanwhile, the temperature distribution of the blank was captured by the infrared ray thermal camera (ε = 0.7, precision: ±2 °C, SC620, FLIR, Wilsonville, OR, USA). The infrared thermal camera was handheld by an operator and is not shown in Figure 1a.

### 2.3. Microstructure Characterization

**Figure 2:**
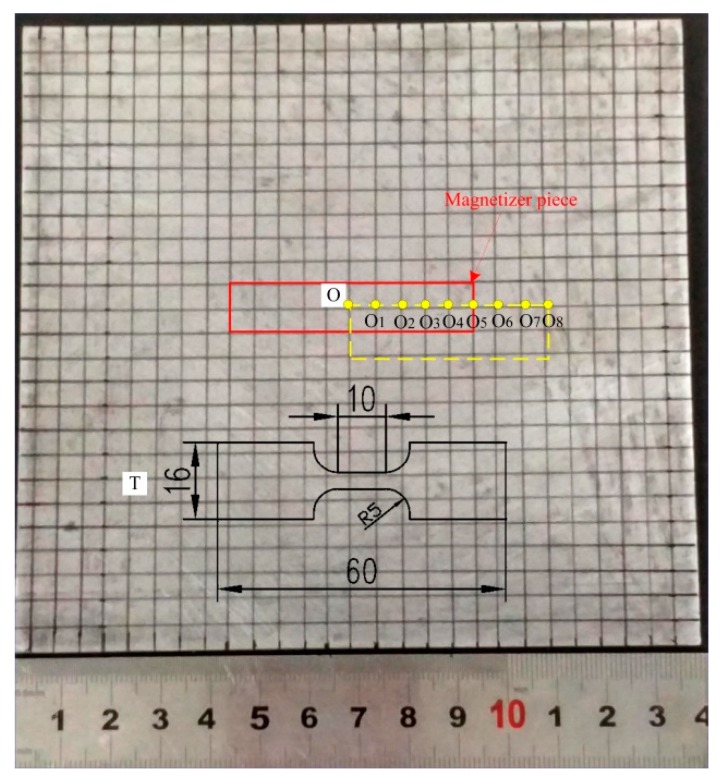
Sample position in the quenched blank. (The red frame: The projection position of the magnetizer piece; O_1_–O_8_ on yellow frame: Metallographic sampling points location; O and T: Tensile specimen size and location).

After a test sample was inductively heated to austenite, it was water quenched to simulate the cooling process during stamping. The microstructures at different locations of the quenched specimen corresponding to two temperature regions were characterized by an optical microscope (OM, LEICA Q550 IW, Wetzlar, Germany) after preparation by standard mechanical grinding procedures and etching in 4% Nital solution. The Rockwell hardness on the upper surface of the quenched specimen was recorded by a Rockwell hardness tester (HR-150A, LLT, Laizhou, China) at a load of 150 N and an interval of 5 mm between the testing points. The data of three samples at each position was recorded and the arithmetic mean was selected as the basis of hardness distribution. The mechanical behavior was characterized using uniaxial tensile tests with three repetitions per microstructure. The blanks were machined into dog-bone specimens with a gauge length equal to 10 mm and a width equal to 3 mm. The tensile tests were performed at room temperature at a nominal strain rate of 0.005/s. The locations of specimens for these tests are shown in Figure 2.

## 3. Results

### 3.1. The Temperature Field of the Steel Blank after Uniform Induction Heating

**Figure 3:**
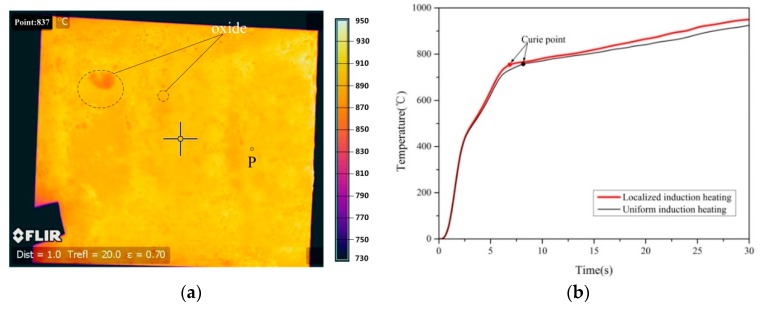
The temperature field and heating history curves of the steel blank due to different heating processes. (**a**) The temperature field with the measured point P and typical oxide location under uniform heating; and (**b**) the comparisons of heating history curves under two heating methods.

Since uniform induction heating is the basis of localized induction heating, the temperature field of the steel blank after 30 s of uniform induction heating and 2 s of transfer is illustrated in Figure 3. Moreover, the heating history profiles of the point P of temperature measurement related to time under uniform heating were compared with the curve under localized induction heating (Figure 3b). Point P was located at the intersection between the place 30 mm from the right edge of the blank and the center line of the blank width, or namely the center of the distance between the second and third copper pipes from the right side. Some representative results from three samples under the same heating process are shown in Figure 3b. Since the samples were treated at the same process conditions, they exhibited low deviations.

A uniform temperature field of the steel blank was induced and the temperature reached 925 °C (Figure 3). The two curves in Figure 3b feature two heating intervals at different heating rates. The inflexion point corresponds to the Curie temperature (T_C_ = 760 °C), which will rise with the increasing heating rate [13]. Moreover, the rate of the localized heating is higher than that of the uniform heating, arrives at T_C_ earlier, and has a higher ultimate temperature (950 °C). The reason is attributed to the effect of the magnetizer piece, which will be clearly explained later. After reaching Tc, the heating rate decreases and is nearly constant for two experiments with values ranging from 7.5 to 8.3 K·s^−1^. This is because, due to the magnetic properties of steel, the heating rate cannot be further raised once the material is paramagnetic [14]. However, the oxide is unevenly distributed on the surface of the uncoated steel blank and the small oxide points are sporadically distributed on the blank, which suggest the oxide may noticeably affect the judgment of temperature distribution of the blank.

When the steel blank was removed from the inductor and transferred to the quenching device, its temperature greatly decreased at a rate of ~40 °C/s. This phenomenon little affected the judgment of the temperature distribution of the uniformly heated steel blank because the distribution was uniform, and most areas of the blank were uniformly reduced except the corners. However, this rapid cooling of the blank greatly influenced its temperature distribution under localized heating. The temperature regions on the steel blank after local heating may undergo different cooling rates and mutually integrate due to heat conduction, so the influence of local heating cannot be well reflected and identified. Therefore, temperature rise and distribution of the steel blank in the inductor would be reasonably observed during the localized induction heating.

### 3.2. Temperature Field of Steel Blank during Localized Induction Heating 

**Figure 4:**
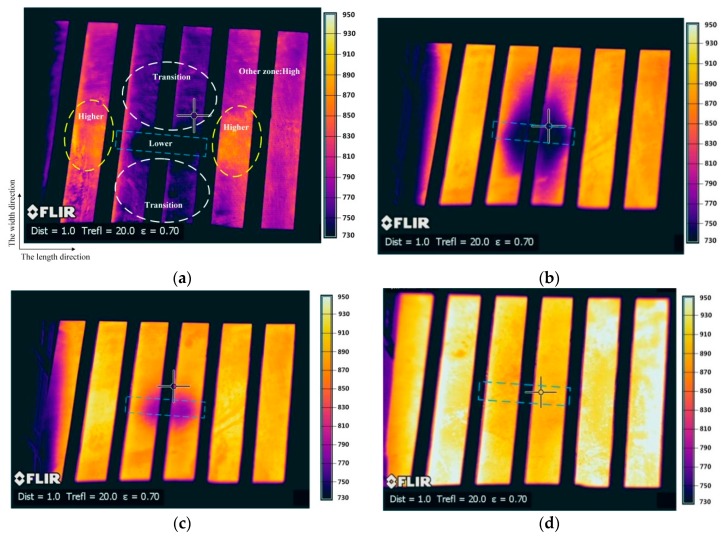
The thermal image of the steel blank during localized induction heating. (**a**) t = 20 s; (**b**) t = 22 s; (**c**) t = 24 s; and (**d**) t = 30 s.

The temperature fields of steel blanks investigated during 30 s of localized induction heating are shown in Figure 4, where the blue frame indicates the projection dimension and position of the magnetizer (which is right under the blank at a 1 mm distance) and the black fence indicates the location of the copper tubes. Moreover, the blank upper surface was heated. In detail, since the blank (140 mm long) was slightly longer than the internal space formed by the solenoid, the left edge of the blank was outside the solenoid, which explains the lower temperature at the left edge. Under the influence of the magnetizer piece, a dark area formed in the center of the steel blank representing the lower-temperature region.

The reason for this phenomenon is attributed to the shielding effect of the magnetizer on the eddy currents induced in the steel blank. The magnetic flux preferentially passed through the magnetizer, due to its higher magnetic permeability, instead of the steel blank which was right above the magnetizer. As a result, the eddy currents induced in this zone were reduced, forming a lower-temperature region. Although the lower-temperature region was obvious, its shape did not exactly match the dimension of the magnetizer piece. The purple dark area partially exceeded the magnetizer width and was named the transition region. Meanwhile, the neighborhood close to both ends of the magnetizer along its length direction was under noticeably higher temperature than at the body of the steel blank and can be named the higher-temperature region. Another region occupying a large proportion on the steel blank was unaffected by the magnetizer and called the high-temperature region. In detail, the positions of different temperature regions are schematically shown in Figure 4a as an example. 

As the temperature exceeded T_C_, the heating rate greatly decreased (Figure 3b), which was favorable for the steel blank under the localized induction heating and was beneficial to avoid overheating in the higher-temperature region. The heat conduction in the blank reduced the transition region. Furthermore, the lower-temperature region was shortened by the higher-temperature region in the length direction and was shorter than the projection length of the magnetizer piece. Figure 4c depicts the approximate outline of the magnetizer piece on the temperature field of the blank at t = 24 s. The higher-temperature region and other high-temperature regions seemed to have merged to reach 950 °C (Figure 4d). However, the oxides started to be gradually formed after 25 s (Figure 4d). Unfortunately, the ranges of only the high-temperature region and the lower-temperature region can be roughly judged, which means the oxides affected to distinguish between different temperature regions in the heated uncoated blank based on the thermal image at t = 30 s. In summary, after 30 s of heating, the temperature obviously minimized to 820 °C in the lower-temperature region and maximized to 950 °C in the high-temperature region and the transition region was generated on the steel blank. It is confirmed that different regions can be generated on the blank by the magnetizer under the localized induction heating. Nevertheless, the history of the time-related temperature distribution and the hardness distribution should be combined in order to clarify the boundaries of different temperature regions eventually formed in the blank.

### 3.3. The Tailored Properties Obtained by Water Quenching the Locally Heated Blank

**Figure 5:**
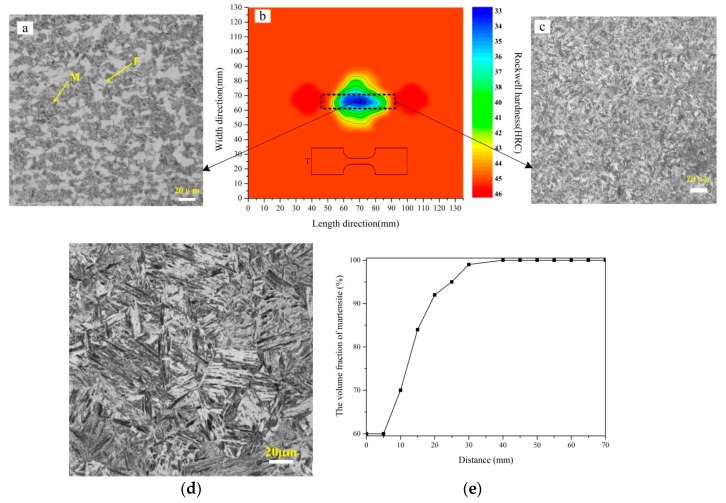
The hardness distribution, optical microscope (OM) images, tensile specimen size and location, the martensite volume fraction. (**a**) Mixture microstructure in the lower-temperature region; (**b**) the hardness distribution and tensile location (O and T); (**c**) martensite in the higher-temperature region; (**d**) martensite obtained by conventional heating–water quenching; and (**e**) martensite volume fraction along the horizontal positive direction from the blank center.

The quenched blank with tailored properties was obtained by localized induction heating and water quenching. The bottom surface of the steel blank was closer to the magnetizer, which more affected different temperature regions, but the temperature distributions at the upper surface and the bottom surface should be similar. Moreover, the hardness on the two surfaces of the quenched blank was measured. It was found that the hardness of the bottom surface was similar to that of the upper surface, and the hardness was lower at the lower-temperature zones and higher at the higher-temperature zones. In order to correspond to the temperature field of the upper surface for temperature measuring, the equal hardness distribution of the upper surface was investigated and is shown in Figure 5b, in which the black frame represents the projection of the magnetizer piece. Moreover, the metallographic microstructures from different temperature regions were taken from the section of the blank every 5 mm along the two centerlines. Typical metallographic microstructures of the quenched blank are depicted in Figure 5a,c, respectively.

The hardness distribution of the quenched blank (Figure 5b) agrees well with the temperature pattern of the blank in Figure 4c. While the temperature at the center of the lower-temperature region (820 °C) corresponds to the Rockwell hardness of 33HRC, and the hardness is more consistent with the high-temperature region occupying the majority of the steel blank than 45HRC. Moreover, the hardness distribution significantly reflects the differential distribution of the temperature field. Obviously, the lower-strength region with higher ductility according to the lower-temperature region is apparently shorter than the magnetizer length. In detail, the lower-temperature region (~35 mm long) is 30% shorter than the projection of the magnetizer. Moreover, the area with the smallest hardness is about 15 × 5 (unit: mm^2^), which will be used to make tensile specimens (Figure 5b). The transition region is about 25 mm wide, compared with the transition region (40 mm) obtained by the mold with differential temperature after the conventional heating [7] or the transition zone (80 mm) of the tailor rolled blanks [15], the transition region (25 mm) obtained in this study is shorter, and it is a gradual transition of the microstructure, which should be applicable for most automotive parts. Furthermore, there are two higher-hardness round zones which may accord with the higher-temperature regions. Fortunately, the hardness is only slightly higher than that according to other high-temperature regions, and the area is smaller.

The typical microstructure image from the lower-temperature regions shows the mixture microstructure consists of ferrite and martensite, whereas the high-temperature region (over 900 °C) is converted to lath martensite after water quenching. The martensite average grain size is less than 10 µm, which is favorable for mechanical properties. Furthermore, on the basis of sampled OM images and the blank center as the origin, the volume fraction of martensite related to distance in the horizontal positive direction as an example was determined on ImageJ. As the distance was prolonged, the temperature gradually rose from the minimum of 820 °C at the center of the lower-temperature region to the maximum of 950 °C (Figure 4d), which means the proportion of austenite and the volume fraction of martensite after quenching also increased with the rise of temperature (Figure 5e).

After taking tensile specimens in the lower-temperature region and at different locations of the high-temperature region, the data from two typical locations were selected (Figure 4b). Therefore, different mechanical properties from the two zones were determined (Table 3). Noticeably, the tensile tests were carried out at various positions in the high-hardness region, and the results were similar to the data in Table 3.

The average ultimate tensile stress (UTS) of the quenched samples related to the lower-temperature region (820 °C) was 977 MPa with high ductility (total elongation 17.5%), but the UTS of the hardened samples reaches 1698 MPa, which is at least 100 MPa higher than the quenched samples after conventional furnace heating and water quenching (Table 3). Due to grain refinement, the strength and ductility of the quenched part can be improved simultaneously. In conclusion, the quenched part with tailored properties can be fabricated by localized induction heating and water quenching. 

## 4. Discussion

After 30 s of localized induction heating, different temperature regions were formed in the steel blank. The variation in quenching temperature led to the difference in strength and ductility in the as-quenched blank for hot stamping.

### 4.1. Reasons for Different Temperature Regions on the Heated Blank

**Figure 6:**
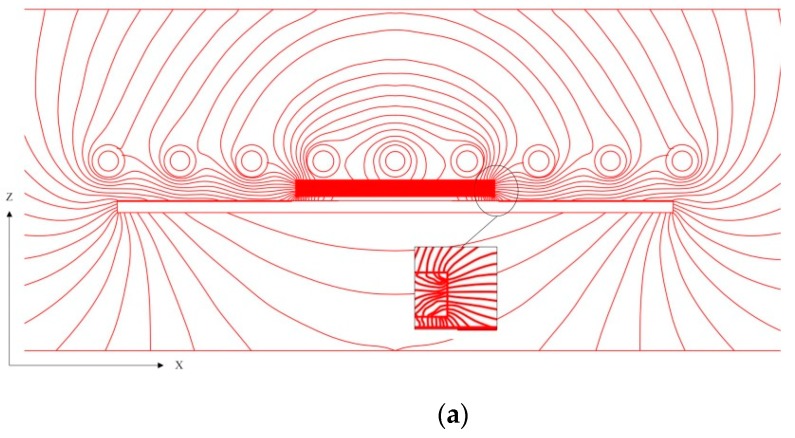

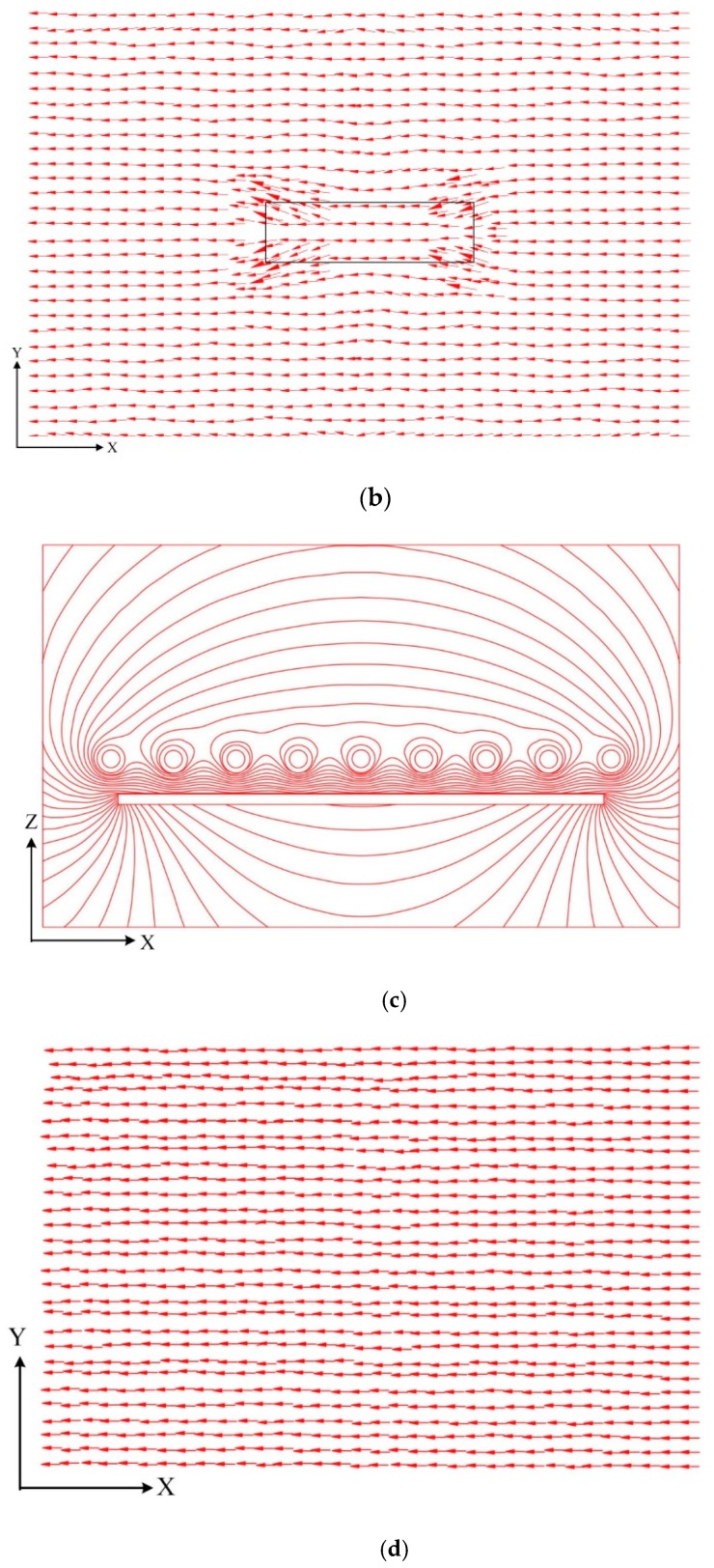
A schematic diagram of the magnetic flux path in two planes with/without a magnetizer piece. (**a**) X–Z plane (cross section of the coils); (**b**) X–Y plane (steel blank surface, the magnetizer depicted as the black frame); (**c**) X–Z plane without magnetizer piece; and (**d**) X–Y plane without magnetizer piece.

Before revealing the designable temperature distribution generated by localized induction heating, the uniform heating of the steel blank within a solenoid should be examined first. The electromagnetic field inside a solenoid is generally uniform. However, as soon as a steel blank enters, the uniformity will be altered to have a denser magnetic flux in the central region of the blank along its length direction. Fortunately, the central concentration is actually balanced by the skin effect at the edge of the workpiece and eventually leads to a uniform inductive heating throughout the entire steel blank. On the basis of uniform induction heating, the localized induction heating will be put forward. A schematic diagram of the magnetic flux path is shown in Figure 6 to easily illustrate the different temperature regions induced on the blank, which is constructed on the basis of the simulation result by the software ANSYS (version 14.5). In order to better understand the change of the magnetic flux field, the schematic diagram of magnetic flux field under uniform heating is also depicted in Figure 6c,d. 

As a magnetizer piece was arranged under the steel blank, the magnetic flux lines preferentially passed through it due to its higher magnetic permeability (Figure 6a). The surrounding magnetic flux entered and passed through different planes of the magnetizer, so the magnetizer was attracted to the surrounding magnetic flux. According to the Gauss theorem of magnetic field, the magnetic flux is positively correlated with the product of magnetic flux density and coverage area [13]. As a consequence, the distribution of eddy current, which is induced by the variation of magnetic flux, determines the temperature distribution.

Due to the low intensity of the magnetic flux lines right under the magnetizer, the eddy current generated was also small and thereby a lower-temperature region was formed. Along the X direction, the magnetic flux lines concentrated near the two ends of the magnetizer and consequently the higher-temperature region was generated, compared with the other high-temperature regions which was unaffected by the magnetizer. The higher-temperature region may be a little different from other high-temperature regions (Figure 4d), which is because this region first reached the Curie temperature (T_C_) and then its heating rate stabilized. The other regions that do not reach the T_C_ were heated up faster, and the heat in the higher-temperature region was also transferred to other temperature regions during this process. Under the comprehensive effect, the temperature difference between the higher-temperature region and other high-temperature regions decreased.

As revealed in the X–Y plane from Figure 6b, due to the sparseness of the magnetic flux lines right below the magnetizer and the attraction of the magnetizer width section in the Y direction, the nearby magnetic flux lines bent toward it, where the magnetic flux density was small. Consequently, the transition region was formed. 

### 4.2. Reasons for Different Strength of the Quenched Part Caused by Differential Temperature

Since the different temperature regions were formed on the steel blank after localized induction heating and after the results of differential strength regions related to the temperature regions were also obtained, the reasons for the generation of these results were then explored. As reported, the start (A_C1_)–finish (A_C3_) temperatures rise with the increasing heating rates according to the TTA-diagram of 22MnB5 steel (Time-Temperature-Austenization) [13] and the A_C1_ and A_C3_ at the heating rate of 100 K·s^−1^ correspond to 750 and 900 °C, respectively [14]. In detail, the heating rate 100 K·s^−1^ corresponds before reaching T_C_, and the rate after T_C_ is 10 K·s^−1^. Since the heating rate (109 K·s^−1^ before T_C_ and 8.3 K·s^−1^ after T_C_) in this study is similar to that of the above research, it is reasonable to estimate that A_C1_ and A_C3_ are about 750 and 900 °C respectively. Therefore, the lower-temperature region with minimum 820 °C on the steel blank obtained here is in the range of the two-phase zone, so the mixed structure of ferrite and martensite can be acquired after water quenching. 

Nevertheless, the high temperature of the lower-temperature region (820 °C) resulted in the high volume fraction of austenite transformed. As a consequence, the martensite volume fraction after quenching was 60% and the temperature of the high-temperature region reached 950 °C, which ensured the complete austenite formation. The fine lath martensite from the high-temperature region was transformed after water quenching. 

### 4.3. Advantages of Localized Induction Heating 

On basis of the results from the localized induction heating, the new local heating process has three advantages:

(1) The time of the localized induction heating (30 s) is only one-tenth that of the conventional heating method, which means the new heating process, has a greatly enhanced heating efficiency and is more energy saving.

(2) In the existing hot stamping production chain, heating is the important step. The new process does not require the change of other process steps, so the cost of improving the original production chain is low. Because of its short heating time, it shortens the overall hot stamping process. These are all beneficial to its future industrial application. 

(3) Since only one magnetizer piece was used to redistribute eddy current induced on the steel blank, the lower-temperature region generated is still small and the temperature is high. On the basis of this result, the tailored temperature region which can customize its pattern, size, and temperature will be investigated in the future by arranging more magnetizers or changing the magnetizer parameters such as size, shape, or permeability. Therefore, the new process is flexible for tailoring the position and pattern of the ductility zones through temperature adjustment.

## 5. Discussion

To meet the lightweight requirements of B-pillar and similar parts with tailored properties, a new localized induction heating process is put forward, in which the high-temperature region on a steel blank is fully austenitized whereas the lower-temperature region is still in the two-phase zone. The water-quenched part with tailored properties is acquired. 

(1) Since the presence of the magnetizer changes the original straight path of the magnetic flux lines and the magnetic flux is redistributed, the distribution of the eddy current induced changes. Therefore, it was experimentally validated and the steel blank with different temperature regions was obtained after 30 s. After water quenching, the high-temperature region of the blank was transformed into fine lath martensite, whereas the lower-temperature region was converted to the ferrite + martensite microstructure. Two enhancement mechanisms for the localized induction heating were realized, including fine-grain strengthening and phase transformation strengthening. The thermal images, hardness distribution, OM images, and mechanical properties of the quenched blanks prove the feasibility and effectiveness of the new localized induction heating process.

(2) The Curie temperature (T_C_) is the inflexion point of the heating rate. The heating rate reaches 109 K·s^−1^ below T_C_ and is greatly slowed above T_C_, which is beneficial to reducing the generation of the overheat zone and thereby is very meaningful for the localized induction heating process.

(3) A_C1_ and A_C3_ rise with the increase of the heating rate. Thus, the start and finish temperatures in the temperature range of the two-phase zone of the new heating process are both higher than those of the conventional heating method, which is consistent with other studies as A_C1_ = 750 °C and A_C3_ = 900 °C.

In summary, the steel blanks with different temperature regions for hot stamping and tailored properties after water quenching can be obtained by the localized induction heating process. The new heating process will meet the industrial flexible and complex requirements as well as the short production cycle of manufacture. 

## Figures and Tables

**Table 1:** Chemical composition of 22MnB5 steel blanks (wt. %).

Chemical Composition	C	Si	Mn	P	S	Cr	Ti	B	Al	Nb
wt. %	0.22	0.24	1.28	0.01	0.003	0.15	0.03	0.003	0.05	0.002

**Table 2:** Physical dimensions.

Item	Parameter	Units
Blank material	22MnB5	-
Blank dimension	140 (L) × 130 (W) × 3.5 (H)	mm
Magnetizer material	Mn–Zn Ferrite	-
Initial relative permeability of magnetizer Magnetizer	400 50 (L) × 10 (W) × 4 (H)	mm
Coil material	Copper	-
Coil dimension	8.5 (outer and inner diameter)	mm
Frequency	100	kHz
Maximum power	100	kW
Total heating time	30	s
Initial and environmental temperature	25	°C
Distance between coil and blank	1	mm
Power supply	SF-C-100	-

**Table 3:** Mechanical properties of hardened samples from different processes. UTS—ultimate tensile stress, YS—yield strength/R_p0.2_.

Process	Heating/ Quenching Temperature	YS	UTS	Total Elongation (%)	Martensite Volume Fraction (%)
(time)	(°C)	(MPa)	(MPa)	-	-
Localized induction heating– water quenching	820	515	977	17.5	60
(heating: 30 s, quenching: 2 min)	950	1320	1698	14.5	100
Conventional heating– water quenching (heating: 5 min, quenching: 2 min)	950	1050	1554	12.0	100
